# Supplementary material for: Global mean sea level likely higher than present during the holocene
Source: Nat Commun. 2024 Dec 30;15:10731. doi: 10.1038/s41467-024-54535-0 (PMC11685612; doi:10.1038/s41467-024-54535-0)
Supplement: Supplementary file 1 — Supplementary Information [file 41467_2024_54535_MOESM1_ESM.pdf]

SUPPORTING INFORMATION FOR: ”GLOBAL MEAN SEA LEVEL HIGHER THAN PRESENT DURING THE HOLOCENE”

Roger C. Creel*[1,2], Jacqueline Austermann[2], Robert Kopp[3], Nicole S. Khan[4], Torsten Albrecht[5], Jonathan Kingslake[1],

1. Department of Physical Oceanography, Woods Hole Oceanographic Institution, Woods Hole, Massachusetts, USA.
2. Lamont-Doherty Earth Observatory, Columbia University, New York, USA.
3. Department of Earth and Planetary Sciences and Rutgers Institute of Earth, Ocean, and Atmospheric Sciences, Rutgers University, New Jersey, USA.
4. Dept of Earth Science and Swire Institute of Marine Science, University of Hong Kong, Hong Kong.
5. Potsdam Institute for Climate Impacts Research, Germany, USA.

*rcreel@ldeo.columbia.edu

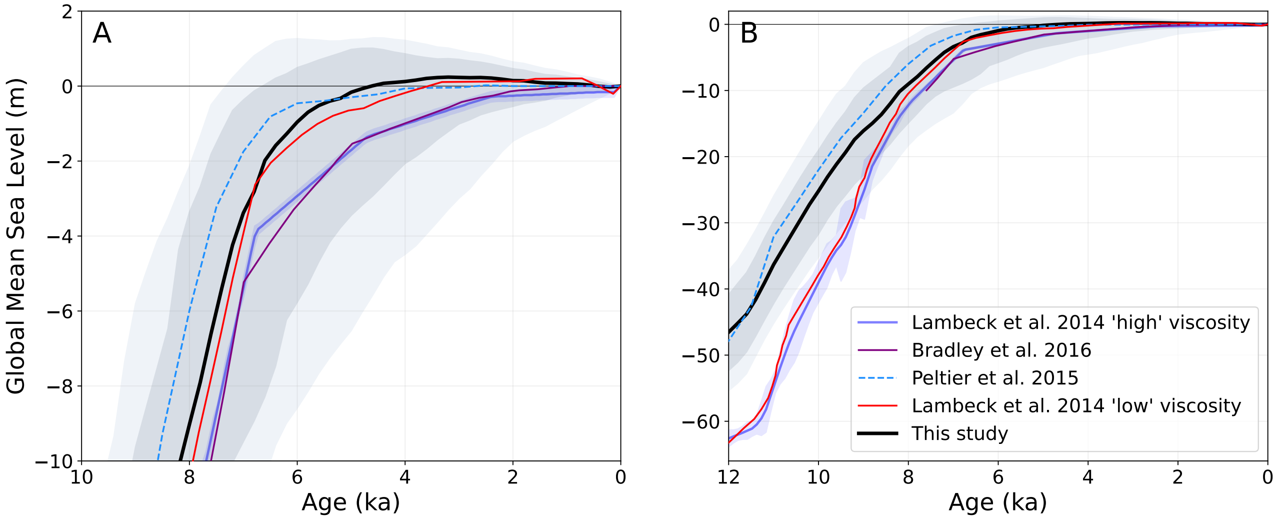

Figure S1: Global mean sea level 8 ka - present (A) and 12 ka - present (B). Green lines and blue lines with 95% credible interval are ice volume equivalent sea level from Peltier and colleagues (Peltier *et al.*, 2015) and Lambeck and colleagues (Lambeck *et al.*, 2014). Red line denotes alternate ice volume equivalent curve from Lambeck and colleagues (Lambeck *et al.*, 2014). Purple lines are ice-volume equivalent sea level from Bradley and colleagues (Bradley *et al.*, 2016), which is corrected for ice above floatation. Black lines with 66% (darker grey) and 90% (lighter grey) credible interval are this study.

	Region	Source reference	Data #
1	Global	Hibbert *et al.* (2016)	136
2	New Zealand	Clement *et al.* (2016) and references therein	206
3	US Atlantic coast	Engelhart & Horton (2012) and references therein	813
4	US Pacific coast	Engelhart *et al.* (2015) and references therein	531
5	Northeastern Florida	Hawkes *et al.* (2016)	25
6	Russian Arctic	Baranskaya *et al.* (2018) and references therein	114
7	Southern Africa	Cooper *et al.* (2018)	59
8	Israel	Dean *et al.* (2019)	107
9	Atlantic coast of Europe	García-Artola *et al.* (2018) and references therein	319
10	Rhine-Meuse Delta	Hijma & Cohen (2019) and references therein	106
11	Southeast Asia, Maldives, India & Sri Lanka	Mann *et al.* (2019) and references therein	527
12	Malay Peninsula	Tam *et al.* (2018) and references therein	95
13	Western Mediterranean	Vacchi *et al.* (2018)	233
14	US Atlantic Coast, Newfoundland	Kemp *et al.* (2018)	785
15	Caribbean	Khan *et al.* (2017)	674
16	Florida	Khan *et al.* (2022)	410
17	US Atlantic & Gulf coasts	Love *et al.* (2016) and references therein	854
18	Southern California & Monterey Bay	Reynolds & Simms (2015) and references therein	180
19	China	Zong (2004) and references therein	235
20	North Australia	Woodroffe (2009) and references therein	81
21	Caribbean & South America	Milne *et al.* (2005)	91
22	British Isles of Scilly	Barnett *et al.* (2020)	110
23	Singapore	Chua *et al.* (2021)	20
24	North Wales, UK	Rushby *et al.* (2019)	39
25	South Georgia, sub-Antarctic	Barlow *et al.* (2016)	9
26	Chile	Garrett *et al.* (2020) and references therein	148
27	South China Sea	Xiong *et al.* (2018)	16
28	Central Pacific	Woodroffe *et al.* (2012)	107
29	Central & Western Mediterranean	Vacchi *et al.* (2021) and references therein	345
30	Global	Hibbert *et al.* (2018) and references therein	721
31	South Korea	Song *et al.* (2018) and references therein	22
32	East China	Xiong *et al.* (2020)	17
33	Australia	Dougherty *et al.* (2019)	5

Table S1: List of source references for standardized relative sea level observations. Source data are provided as a Source Data file.

#	Region	Source reference	Data #
34	Australia	Lewis *et al.* (2013) and references therein	350
35	Indonesia	Bender *et al.* (2020)	20
36	Central South Pacific	Hallmann *et al.* (2020)	78
37	French Polynesia	Hallmann *et al.* (2018)	98
38	Nile Delta	Marriner *et al.* (2012)	86
39	South Australia	Belperio *et al.* (2002)	212
44	Ryukyu, Japan	Yokoyama *et al.* (2016) and references therein	15
45	Philippines	Miklavič *et al.* (2018)	10
46	Iriomote Island, Japan	Yamano *et al.* (2019)	15
47	Southeast Australia	Sloss *et al.* (2007) and references therein	176
48	Western Japan	Tanigawa *et al.* (2013)	32
49	Great Barrier Reef, Australia	Leonard *et al.* (2018)	94
50	Great Barrier Reef, Australia	Salas-Saavedra *et al.* (2018)	89
51	Society Islands, Pacific	Gischler *et al.* (2016)	31
52	Brazil	Dechnik *et al.* (2019)	61
53	Río de la Plata, South America	Prieto *et al.* (2017) and references therein	56
54	Brazil	Angulo *et al.* (2018)	9
55	Malay Peninsula	Zhang *et al.* (2021)	14
56	Mekong river delta, Vietnam	Ta *et al.* (2021) and references therein	16
57	Brazil	Angulo & Lesso (1997)	39
58	Tanzania	Punwong *et al.* (2018)	16
59	Beaufort Sea	O'Regan *et al.* (2018)	8
60	Namibia	Runds *et al.* (2019)	6
61	Namibia	Kirkpatrick *et al.* (2019)	7
62	Sardinia	Deiana *et al.* (2021)	2
63	Iberian margin	Leorri *et al.* (2013)	11
64	NE Adriatic Sea	Brunović *et al.* (2020)	8
65	Tunisia	Pleuger *et al.* (2019)	30
66	Tunisia	Khadraoui *et al.* (2019) and references therein	18
67	Society Islands, Pacific	Gischler *et al.* (2019)	24
68	NE Adriatic Sea	Kaniewski *et al.* (2021) and references therein	43
69	Western Mediterranean	Vacchi *et al.* (2020)	18
70	Tierra del Fuego, Chile	Björck *et al.* (2021) and references therein	83
71	Gilbert Islands, Pacific	Yamano *et al.* (2017)	13
72	Marshall Islands, Pacific	Kench *et al.* (2014)	8
73	Cook Islands, Pacific	Gray & Hein (2005)	32
74	Rio de Janiero	Castro *et al.* (2014)	9
75	Zanzibar	Punwong *et al.* (2013); Punwong (2013)	3
76	Bonaparte Gulf, Australia	De Deckker & Yokoyama (2009)	5
77	Russian Island, Sea of Japan	Grebennikova *et al.* (2020)	1
78	Bangladesh	Rashid *et al.* (2013)	13
79	Sri Lanka	Ratnayake *et al.* (2017)	4

Table S2: List of Source references for additional published relative sea level data. Source data are provided as a Source Data file.

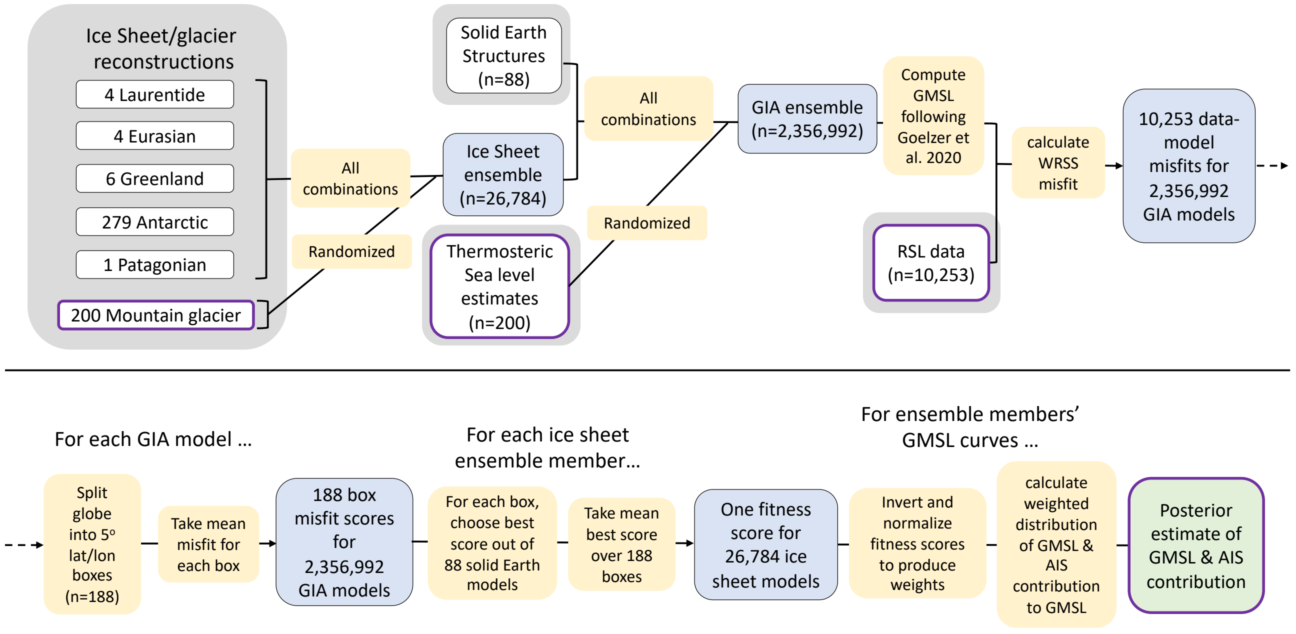

Figure S2: Flow chart depicting the algorithm used to estimate Holocene global mean sea level and ice sheet volume. Grey indicates the introduction of modeling products or data to the algorithm; yellow indicates modeling actions; blue indicates intermediary products; green indicates final product. Purple outlines indicate data and modeling products newly compiled or produced for this study.

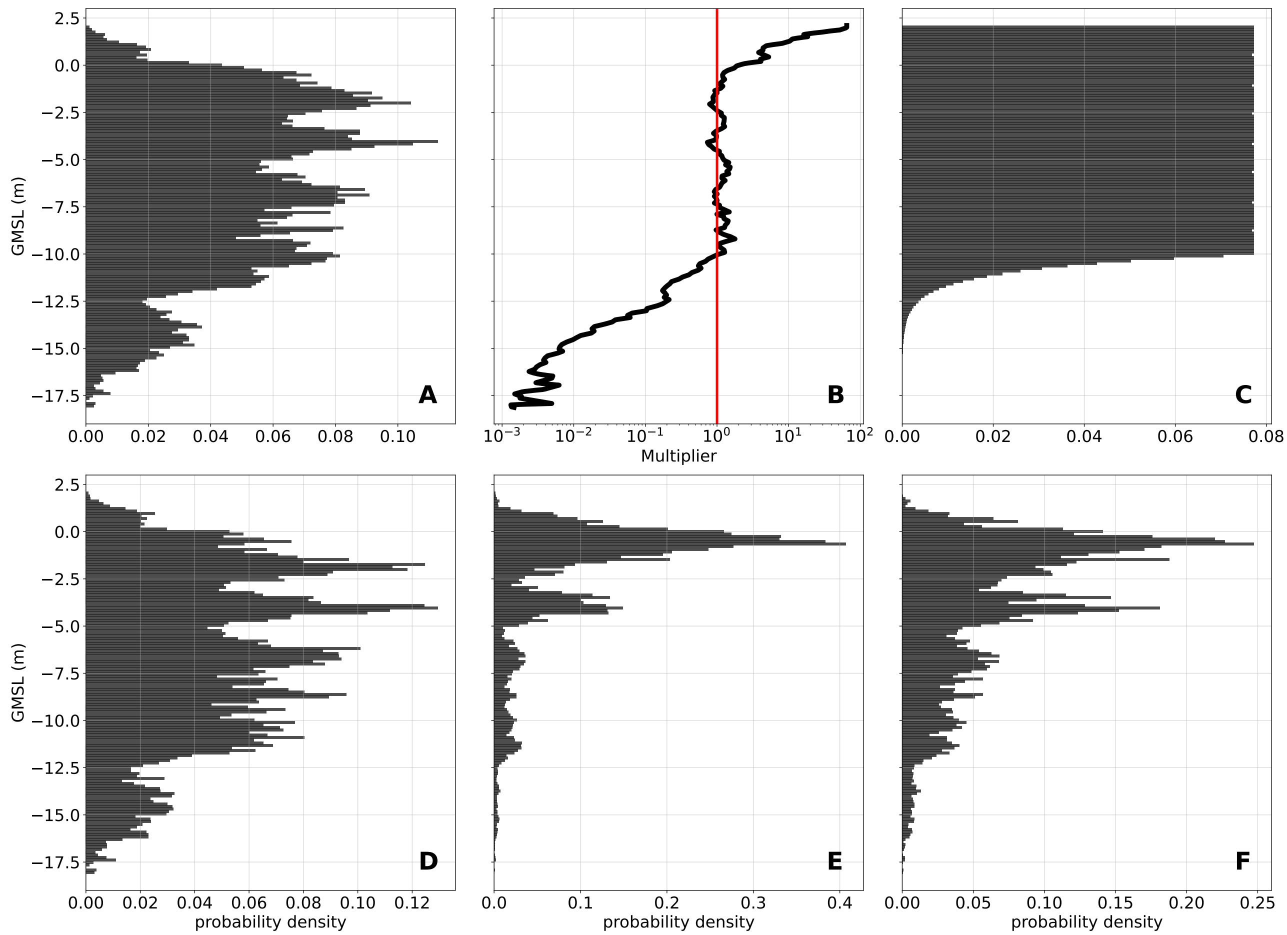

Figure S3: Modeled distributions of global mean sea level (GMSL) in the mid-Holocene (6 ka) demonstrating how data-derived weights are combined to generate the posterior distribution. (A) GMSL distribution with each ice-sheet model assigned equal weight. (B) Weight applied to GMSL curves so that they have even probability density between -10 and +2 m and asymptotically decreasing density below -10 m. Red line denotes a correction factor of one, i.e. no correction applied. Note that a log scale is used on the x axis. (C) Prior GMSL distribution. (D) Posterior GMSL distribution using only weights derived from relative sea level (RSL) data. The difference between (A) and (D) represents the influence of RSL data. (E) Posterior GMSL distribution using only weights formed from Antarctic Ice Sheet fitness scores from Albrecht *et al.* (2020a,b); Albrecht (2019). The difference between (A) and (E) represents the influence of Antarctic Ice Sheet near-field data. (F) Posterior model distribution produced by applying RSL weights and Antarctic Ice Sheet fitness scores to the uniform prior GMSL distribution from (C). The difference between (A) and (F) represents the combined influence of RSL data and Antarctic Ice Sheet fitness scores on our prior (B).

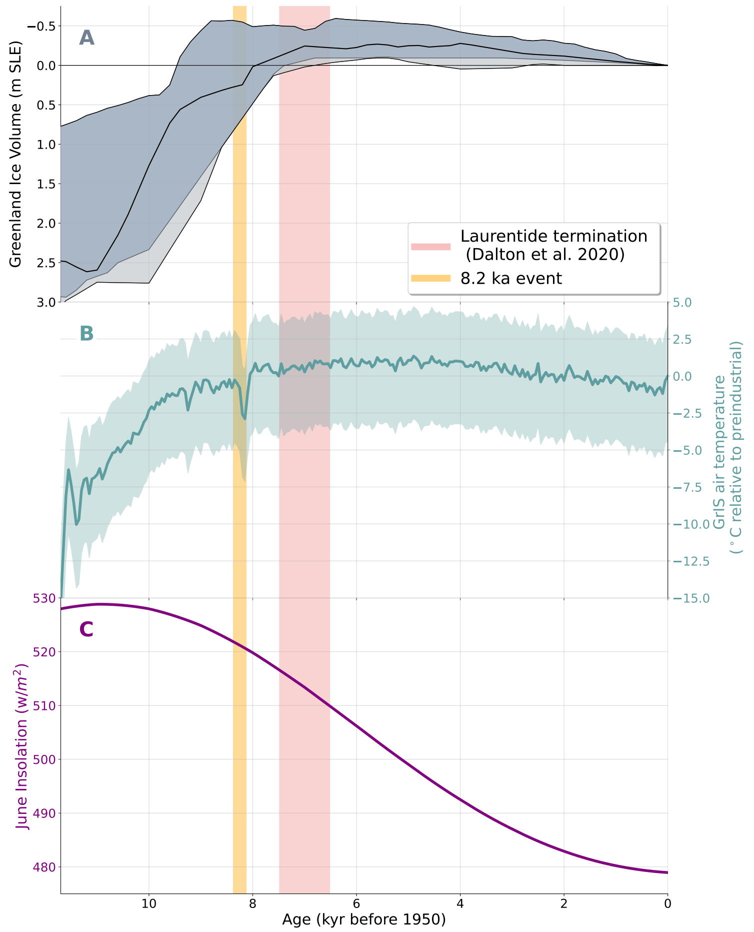

Figure S4: Holocene Greenland ice volume compared to climate variables. (A) Greenland ice volume. Black line denotes posterior 50th quantile; light gray band the posterior 95% credible interval; and darker gray band the posterior 66% credible interval. Prior mean and credible intervals (not shown) are identical to the posterior. (B) Greenland Ice Sheet surface air temperature data assimilation product (Badgeley *et al.*, 2020). Green envelope is 95% confidence interval. Green line denotes mean. (C) June insolation at 65° North. Pink and orange vertical lines indicate final Laurentide termination (Dalton *et al.*, 2020) and the 8.2 ka event, respectively.

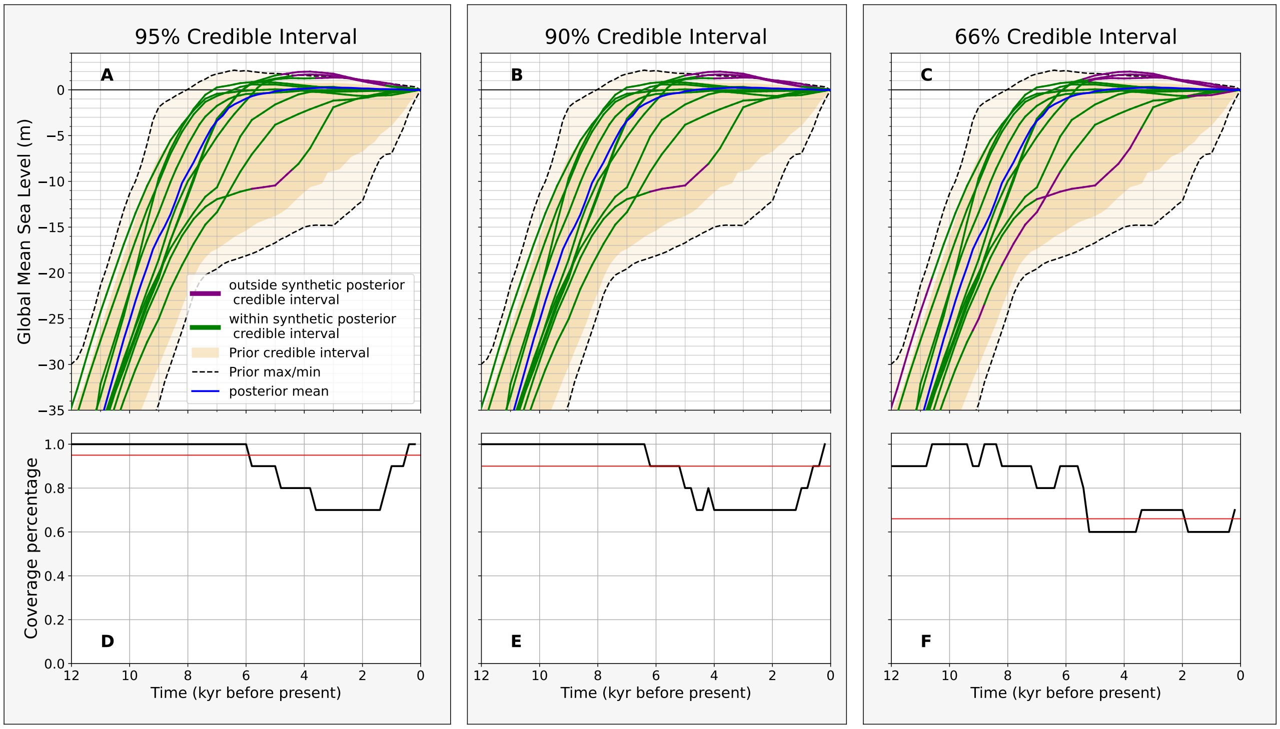

Figure S5: Results of synthetic tests of statistical analysis algorithm. (A) Tan envelope denotes 95% credible interval of prior global mean sea level (GMSL) ensemble. Green/purple Lines trace the 'true' GMSL curves used to generate synthetic relative sea level data, with color denoting time steps where the 'true' GMSL curve is (green) or is not (purple) within the credible interval of the posterior GMSL distribution (see methods). Blue line is the median posterior curve from Fig. 2. (B-C) Identical to (A) but with, respectively, a 90% and 66% credible interval. (D-F) Coverage percentage, i.e. percentage of posterior models in (A-C) whose credible interval successfully captures the associated 'true' synthetic GMSL curve. Red line indicates approximate percentage cutoff considered successful for each interval, e.g. 95% of 'true' curves should fall within the 95% credible interval of the synthetic posterior.

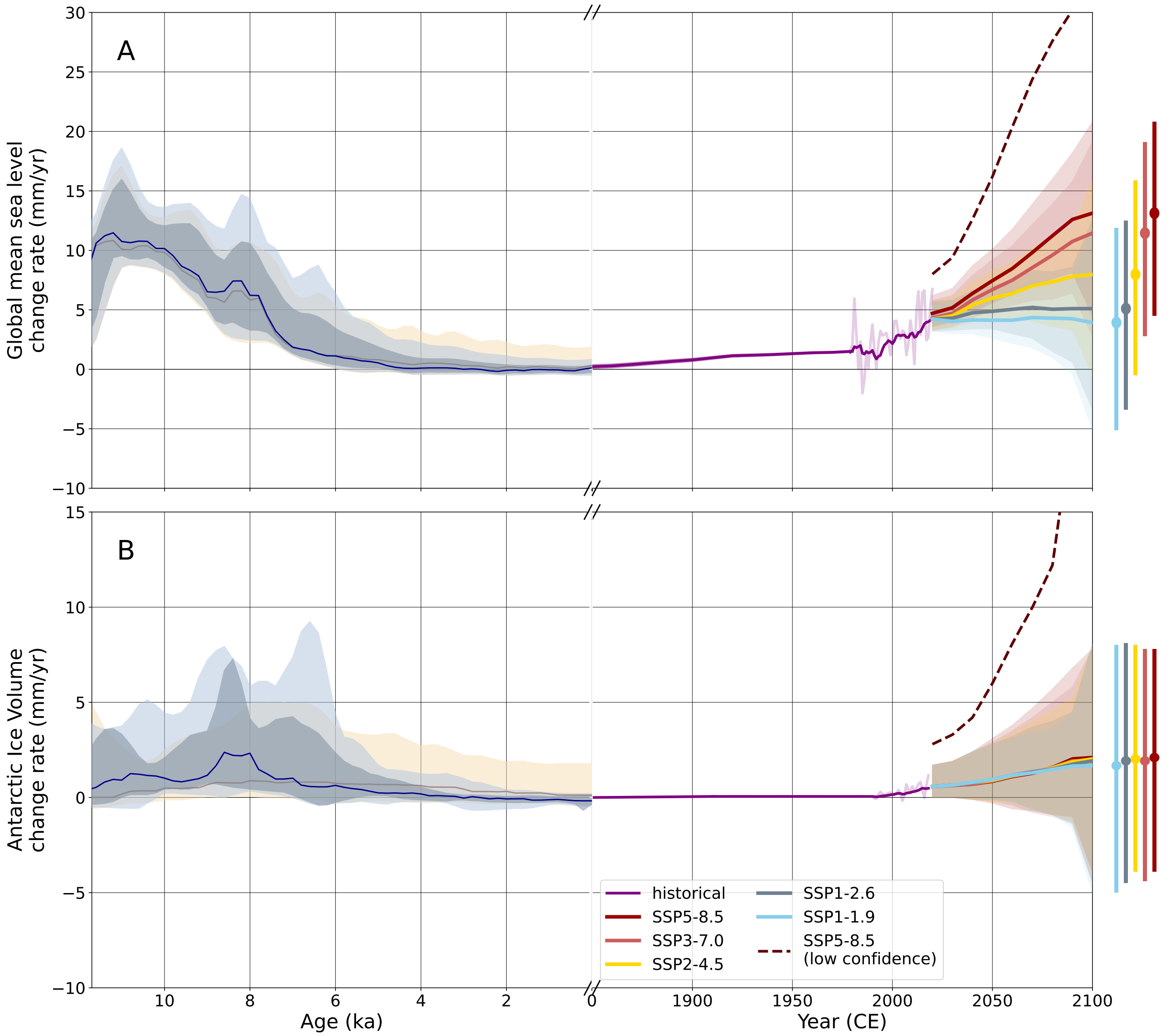

Figure S6: Rates of global mean sea level and Antarctic ice volume change 12 ka - 2100 CE. (A) Rates of global mean sea level change. (B) Rates of Antarctic Ice Sheet volume change. Rates prior to 1850 are from this study. Historical rates 1850 to 1950 are from ref.Walker *et al.* (2022) (A) and ref.Frederikse *et al.* (2020) (B). Rates 1950 to 2100 are 66% credible intervals from the Intergovernmental Panel on Climate Change's 6th Assessment Report (IPCC AR6) (Fox-Kemper, B. *et al.*, 2021). See Figs. 2 and 4 for further details.

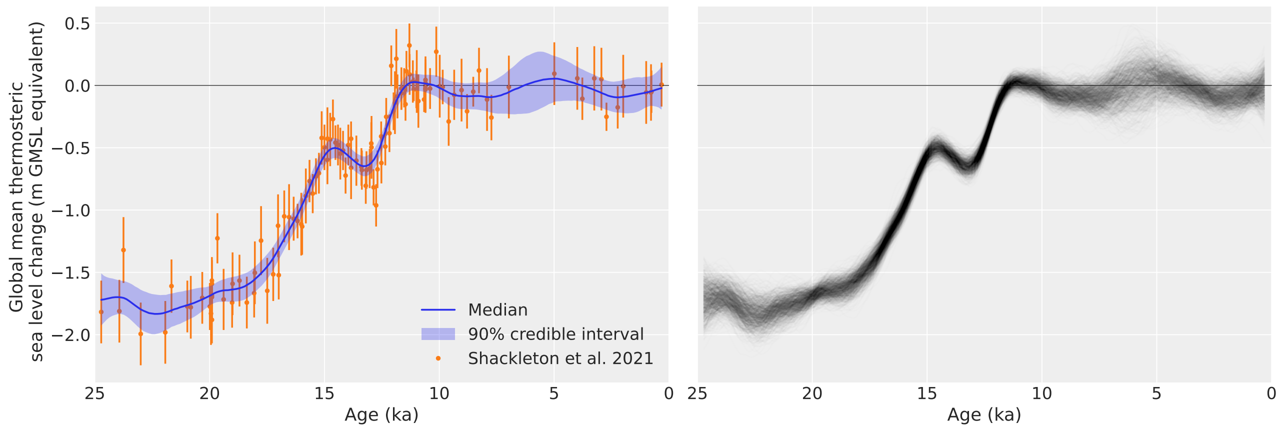

Figure S7: Thermosteric sea-level change 25 ka - present in meters global mean sea level (GMSL) equivalent. Orange markers denote thermosteric sea-level estimates derived from (Shackleton *et al.*, 2021). Blue envelope indicates 90% credible interval derived from Gaussian process regression fit to empirical estimates. Black lines are random draws from posterior distribution.

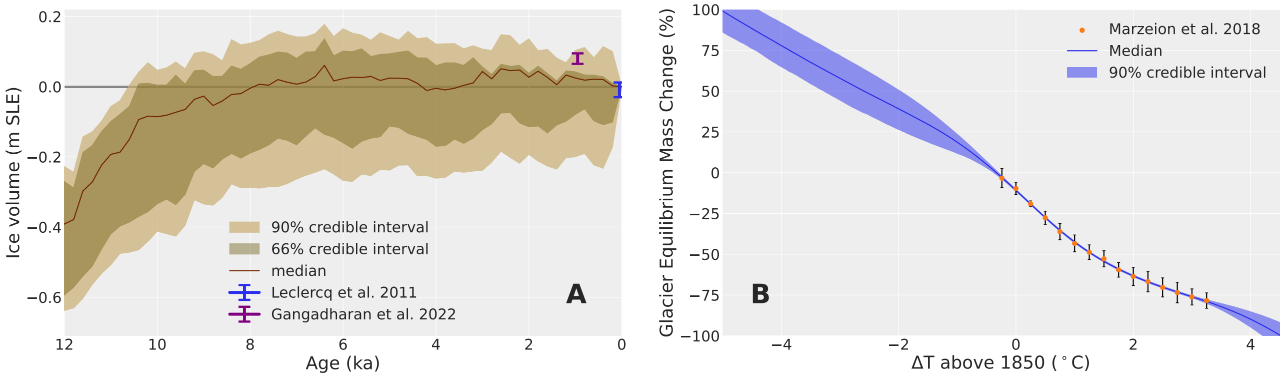

Figure S8: Mountain glacier posterior volume in global mean sea level-equivalent units. (A) Light brown indicates 90% credible interval; darker brown denotes 66% credible interval. Prior volume, not shown, is identical to posterior volume. Purple and blue error bars denote max/min empirical estimates of the mountain glacier contribution to global mean sea level from ref. (Gangadharan *et al.*, 2022) and ref. (Leclercq *et al.*, 2011), respectively. (B) Mountain glacier equilibrium mass change per degree of temperature change relative to 1850. Orange dots denote empirical mass change - temperature scaling relations(Marzeion *et al.*, 2018). Blue envelope indicates 90% credible interval from Gaussian process regression fit to extended empirical estimates.

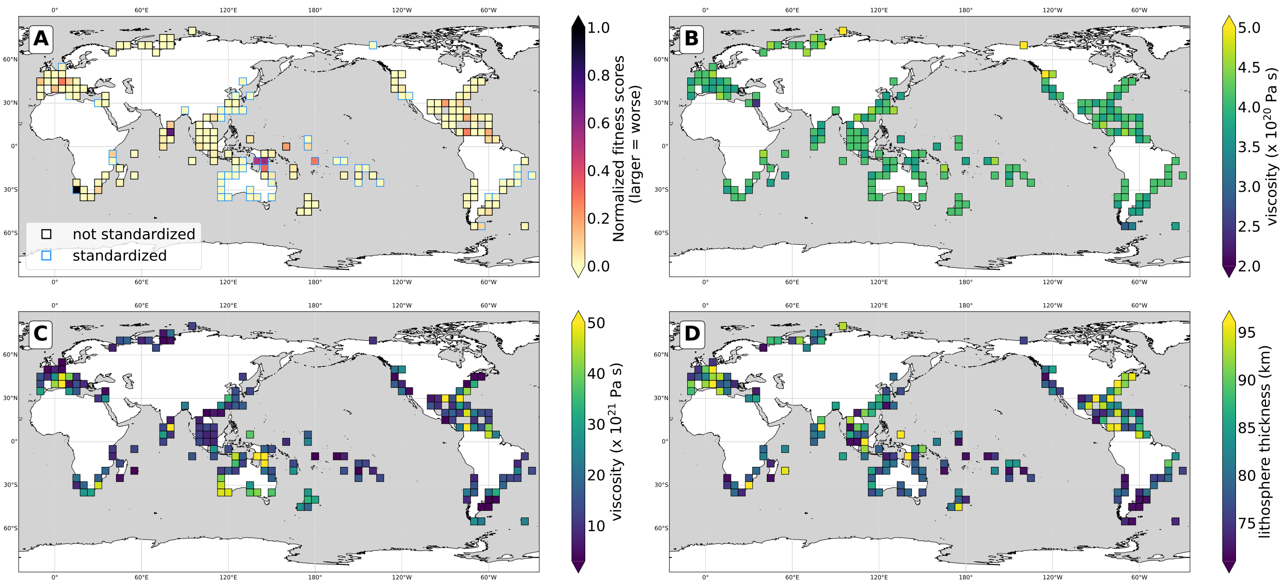

Figure S9: Fitness scores from statistical analysis. (A) Normalized weighted mean of fitness metrics for each site. Black box indicates that the majority of RSL observations at that site are standardized; Blue box indicates not standardized. (B) Upper mantle viscosities. (C) Lower mantle viscosities. (D) Lithospheric thickness.

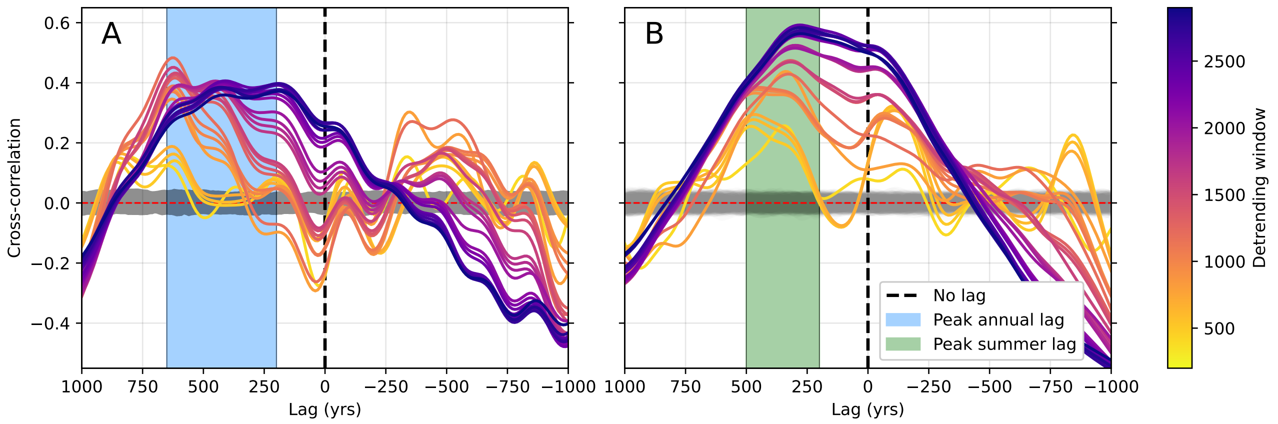

Figure S10: Cross-correlation between West Antarctica surface air temperature records and Antarctic ice volume. (A) Cross-correlations between mean annual air temperatures from the West Antarctic Ice Sheet Divide ice core (Cuffey *et al.*, 2016) and median Antarctic ice volumes (this study). Detrending windows from 400 to 3000 years (colorbar) are applied. Significance (light grey bars) is estimated via a 500 member ensemble analysis; Correlation segments that are outside the grey bar are considered significant. See ref. (Rehfeld *et al.*, 2011; Rehfeld & Kurths, 2014) for more details. (B) Cross-correlations between mean West Antarctic Ice Sheet Divide summer air temperatures (Jones *et al.*, 2023) and median Antarctic ice volumes. Blue and green bars respectively indicate periods of peak annual and summer lag. Black dashed line marks zero lag.

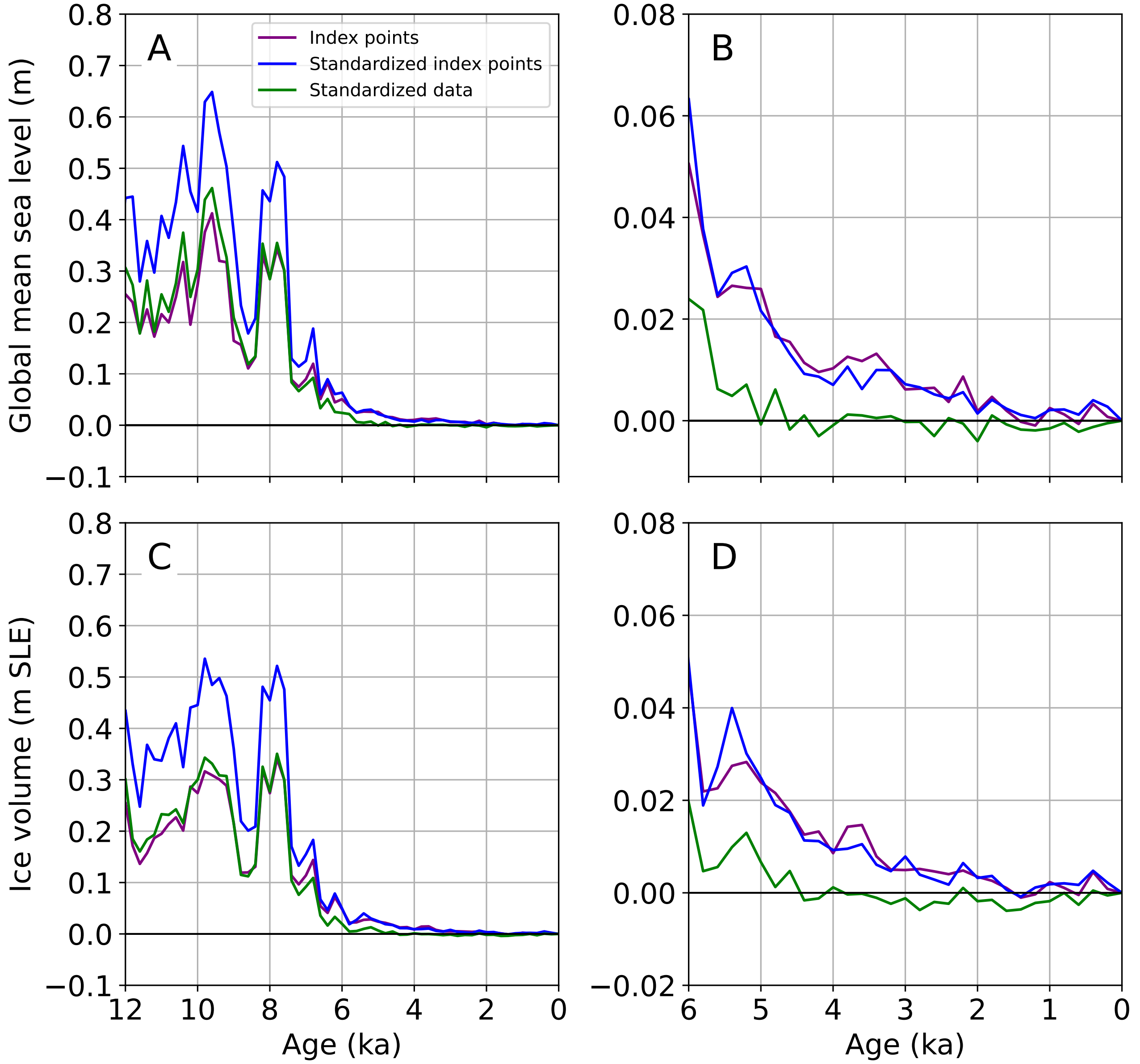

Figure S11: Sensitivity tests to explore how using subsets of the full relative sea level (RSL) dataset affect our results. (A/B) Difference between median posterior global mean sea level (GMSL) when all data are included in the model versus when only a subset is included (i.e., inference from all data minus inference from subset of data). Subsets are only index points (purple), only index points standardized following agreed-upon community conventions for data quality (Khan *et al.*, 2019)(blue), and only standardized data including marine- and terrestrial-limiting points (green). B is a zoom-in of A. (C/D) Differences between median posterior Antarctic ice sheet contribution to barystatic sea level when all data are included in the model versus when only a subset is included. Subsets are the same as in (A/B). Units are global mean sea level equivalent (SLE).

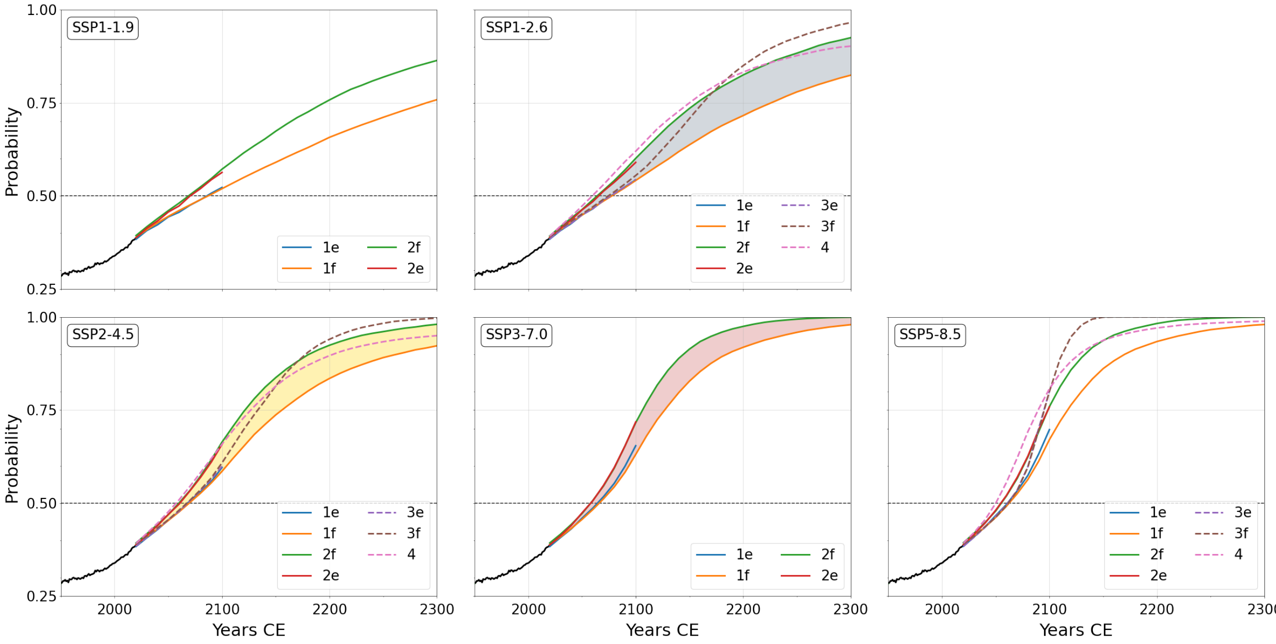

Figure S12: Exceedance probabilities for each global mean sea level (GMSL) workflow from the Intergovernmental Panel on Climate Change's 6th Assessment Report (IPCC AR6). Colored lines denote individual IPCC AR6 workflows (i.e. 1e, 1f, 2e, 2f, 3e, 3f, 4) that incorporate processes about which there is medium (solid lines) or low (dashed line) confidence. Colored envelopes represent p-box distributions based on medium-confidence workflows. Exceedance probability describes the probability that future GMSL exceeds maximum Holocene GMSL. Workflows are described in ref. (Kopp *et al.*, 2023).

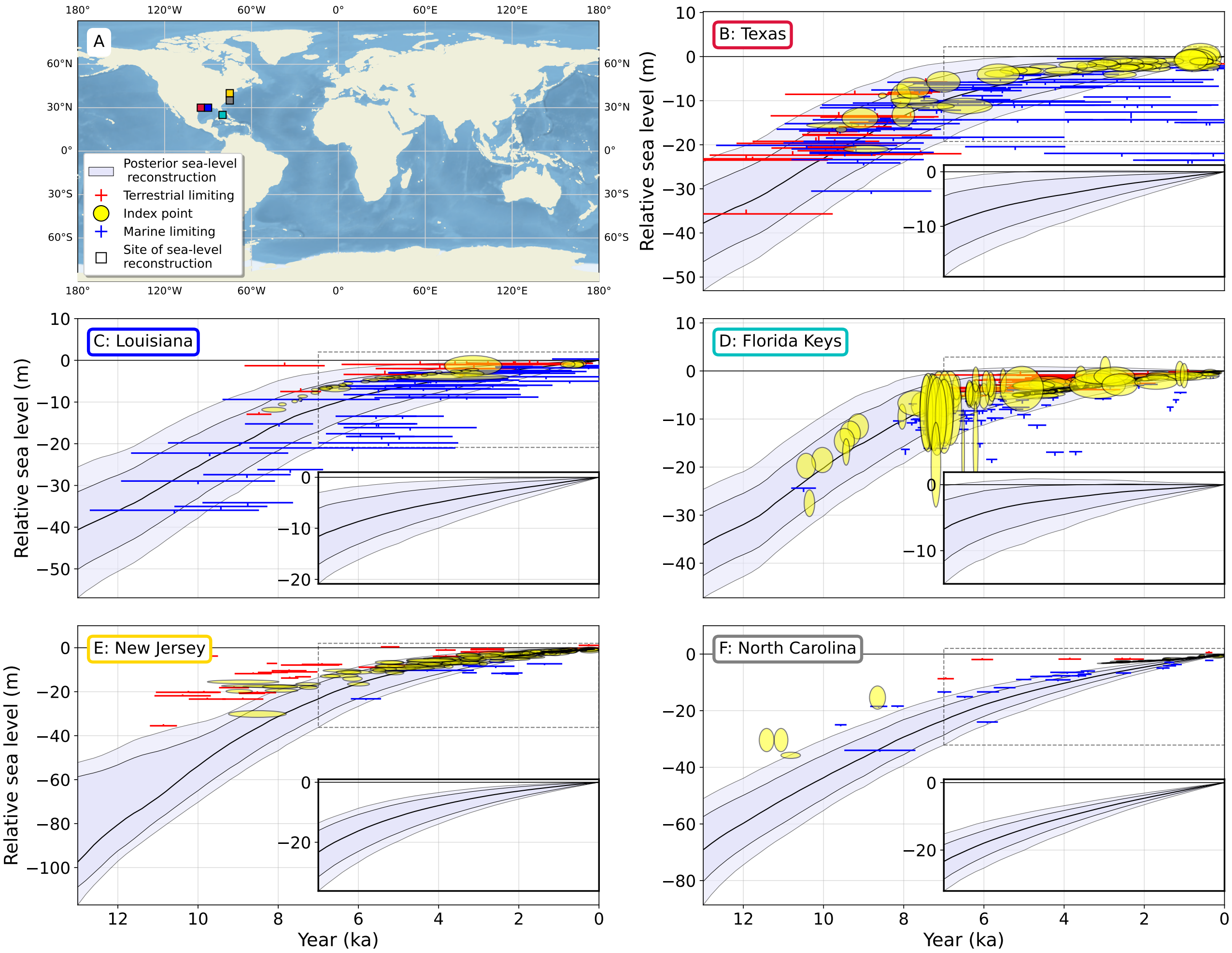

Figure S13: Modeled relative sea-level curves and proxy data for the US East Coast, Gulf Coast, and the Caribbean. (A) Colored boxes mark locations of relative sea-level (RSL) curves in (B - F). (B-F) Black lines with lavender envelopes are mean, 66%, and 90% confidence intervals for RSL curves at the center point of the five degree latitude/longitude boxes in (A). Yellow circles and red/blue markers denote index points and terrestrial/marine limiting data from within the boxes in (A); x/y diameters of circles denote 2σ uncertainties. Plotted sea-level data that are standardized following the HOLSEA working group's protocols (Khan *et al.*, 2019) are denoted with stars: (B) Love *et al.* (2016)*; (C) Love *et al.* (2016)*; (D) Khan *et al.* (2017)*, Khan *et al.* (2022)*, Ashe *et al.* (2022)*, Hibbert *et al.* (2018)*; (E) Engelhart & Horton (2012)*, Kemp *et al.* (2018)*; (F) Engelhart & Horton (2012)*, Kemp *et al.* (2018)*.

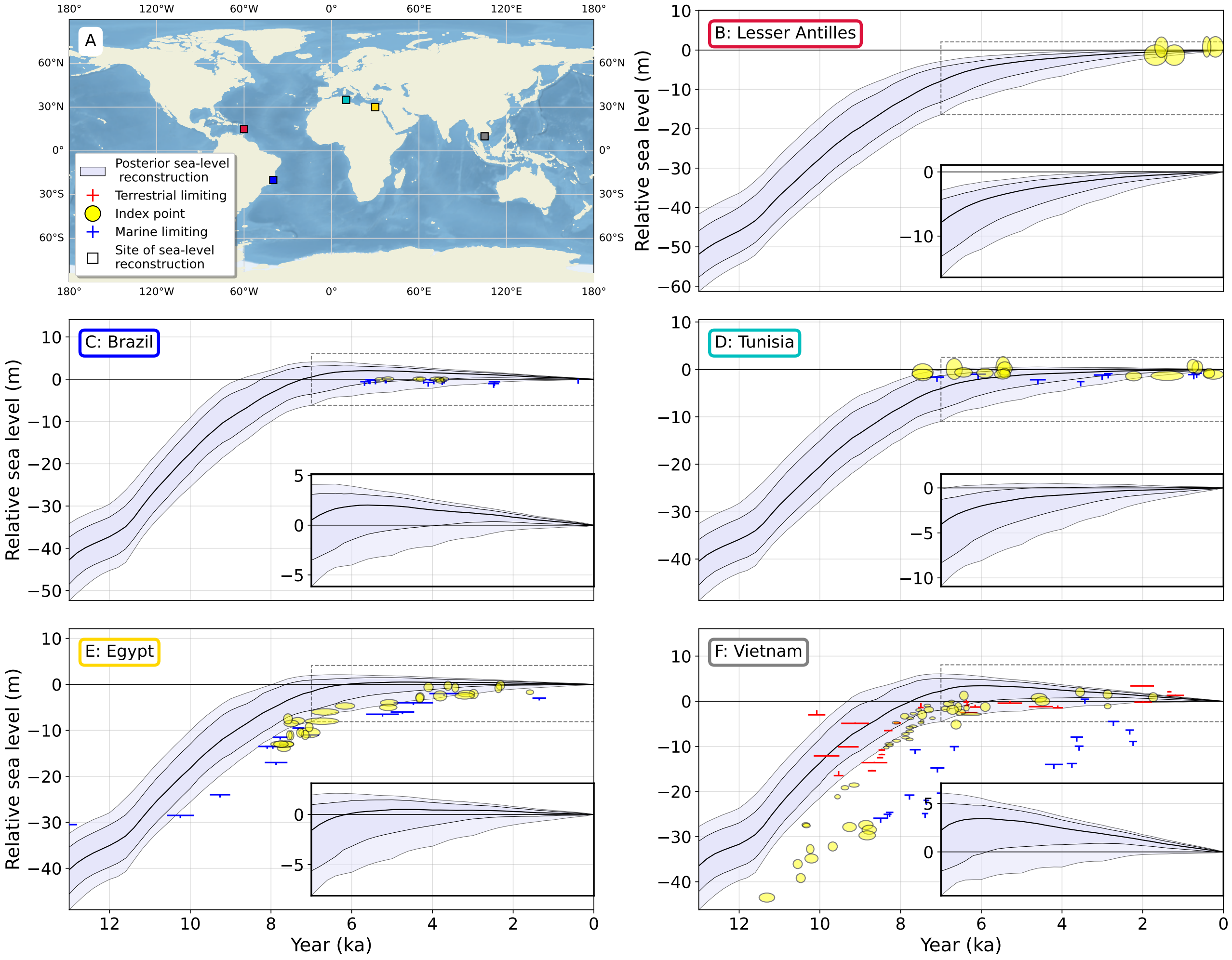

Figure S14: Modeled relative sea-level curves and proxy data for the Caribbean, South America, Mediterranean, and Southeast Asia. Sea-level data are from: (B) Khan *et al.* (2017)*; (C) Dechnik *et al.* (2019)*; (D) Vacchi *et al.* (2021)*, Khadraoui *et al.* (2019); (E) Marriner *et al.* (2012); (F) Mann *et al.* (2019)*, Ta *et al.* (2021); See Supplemental Figure S13 for more information.

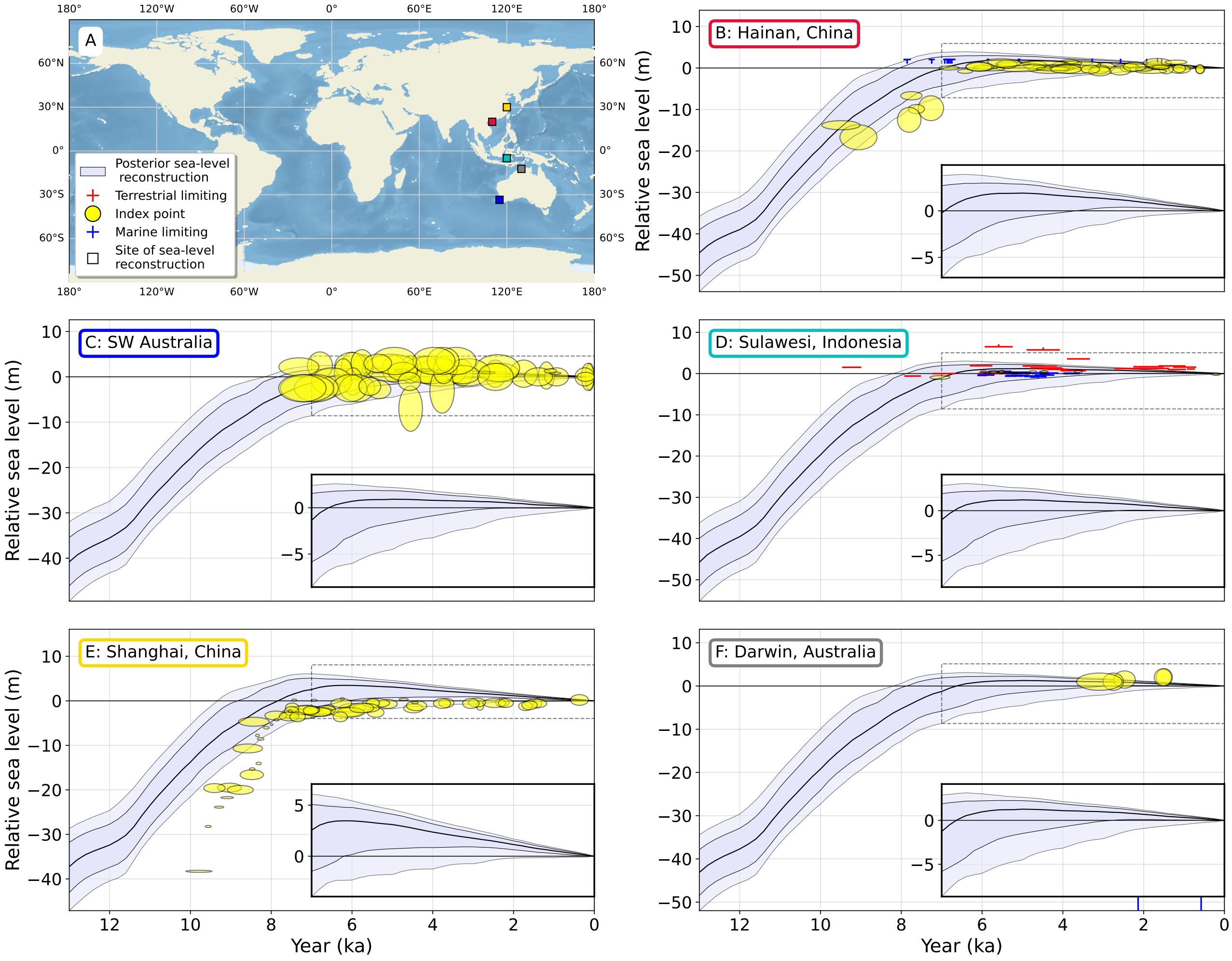

Figure S15: Modeled relative sea-level curves and proxy data for Southeast Asia and Western Australia. Sea-level data are from: (B) Hibbert *et al.* (2016)*, Zong (2004); (C) Lewis *et al.* (2013); (D) Mann *et al.* (2019)*, Bender *et al.* (2020)*; (E) Zong (2004), Xiong *et al.* (2020)*; F Hibbert *et al.* (2018)*, Nott (1996). See Supplemental Figure S13 for more information.

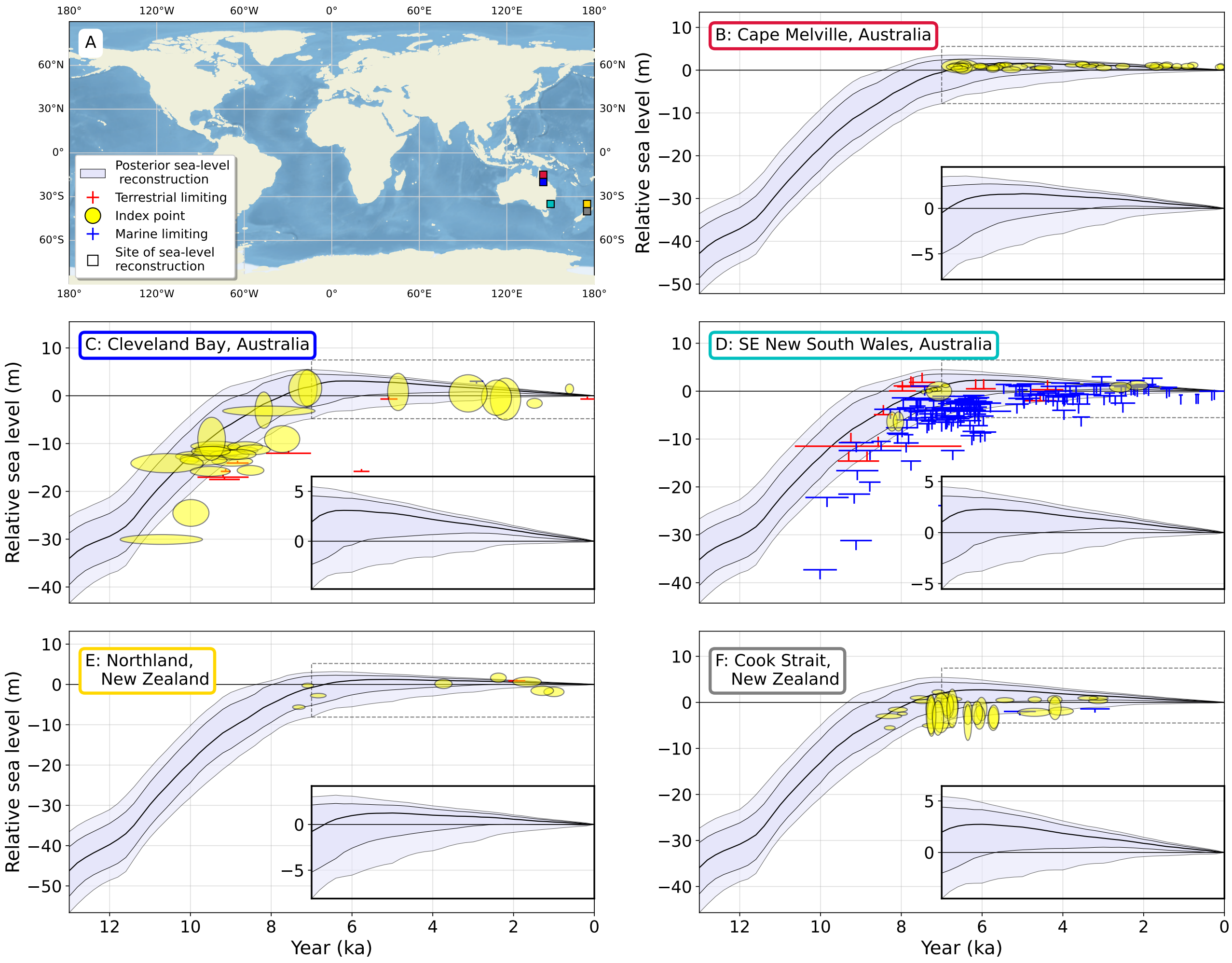

Figure S16: Modeled relative sea-level curves and proxy data for Eastern Australia and New Zealand. Sea-level data are from: (B) Hibbert *et al.* (2018)*, Zwartz (1995), Higley (2000); (C) Woodroffe (2009)*; (D) Sloss *et al.* (2007), Hibbert *et al.* (2018)*, Dougherty *et al.* (2019); (E) Clement *et al.* (2016)*; (F) Clement *et al.* (2016)*, Hibbert *et al.* (2018); See Supplemental Figure S13 for more information.

References

Albrecht, T., 2019. PISM glacial cycle sensitivity experiments of the Antarctic Ice Sheet.

Albrecht, T., Winkelmann, R., & Levermann, A., 2020. Glacial-cycle simulations of the Antarctic Ice Sheet with the Parallel Ice Sheet Model (PISM) – Part 2: Parameter ensemble analysis, *The Cryosphere*, **14**(2), 633–656.

Albrecht, T., Winkelmann, R., & Levermann, A., 2020. Glacial-cycle simulations of the Antarctic Ice Sheet with the Parallel Ice Sheet Model (PISM) – Part 1: Boundary conditions and climatic forcing, *The Cryosphere*, **14**(2), 599–632.

Angulo & Lesso, 1997. The Brazilian sea-level curves: A critical review with emphasis on the curves from the Paranaguáand Cananéia regions, *Marine Geology*, **140**(1-2), 141–166.

Angulo, R. J., de Souza, M. C., Barboza, E. G., Rosa, M. L. C. d. C., Fernandes, L. A., Guedes, C. C. F., de Oliveira, L. H. S., Manzolli, R. P., Disaró, S. T., Ferreira, A. G., & Martin, C. M., 2018. Quaternary sea-level changes and coastal evolution of the Island of Trindade, Brazil, *Journal of South American Earth Sciences*, **84**, 208–222.

Ashe, E. L., Khan, N. S., Toth, L. T., Dutton, A., & Kopp, R. E., 2022. A statistical framework for integrating nonparametric proxy distributions into geological reconstructions of relative sea level, *Advances in Statistical Climatology, Meteorology and Oceanography*, **8**(1), 1–29.

Badgeley, J. A., Steig, E. J., Hakim, G. J., & Fudge, T. J., 2020. Greenland temperature and precipitation over the last 20,000 years using data assimilation, *Climate of the Past*, **16**(4), 1325–1346.

Baranskaya, A. V., Khan, N. S., Romanenko, F. A., Roy, K., Peltier, W. R., & Horton, B. P., 2018. A postglacial relative sea-level database for the Russian Arctic coast, *Quaternary Science Reviews*, **199**, 188–205.

Barlow, N. L. M., Bentley, M. J., Spada, G., Evans, D. J. A., Hansom, J. D., Brader, M. D., White, D. A., Zander, A., & Berg, S., 2016. Testing models of ice cap extent, South Georgia, sub-Antarctic, *Quaternary Science Reviews*, **154**, 157–168.

Barnett, R. L., Charman, D. J., Johns, C., Ward, S. L., Bevan, A., Bradley, S. L., Camidge, K., Fyfe, R. M., Gehrels, W. R., Gehrels, M. J., Hatton, J., Khan, N. S., Marshall, P., Maezumi, S. Y., Mills, S., Mulville, J., Perez, M., Roberts, H. M., Scourse, J. D., Shepherd, F., & Stevens, T., 2020. Nonlinear landscape and cultural response to sea-level rise, *Science Advances*, **6**(45), eabb6376.

Belperio, A. P., Harvey, N., & Bourman, R. P., 2002. Spatial and temporal variability in the Holocene sea-level record of the South Australian coastline, *Sedimentary Geology*, **150**(1), 153–169.

Bender, M., Mann, T., Stocchi, P., Kneer, D., Schöne, T., Illigner, J., Jompa, J., & Rovere, A., 2020. Late Holocene (0–6 ka) sea-level changes in the Makassar Strait, Indonesia, *Climate of the Past*, **16**(4), 1187–1205.

Björck, S., Lambeck, K., Möller, P., Waldmann, N., Bennike, O., Jiang, H., Li, D., Sandgren, P., Nielsen, A. B., & Porter, C. T., 2021. Relative sea level changes and glacio-isostatic modelling in the Beagle Channel, Tierra del Fuego, Chile: Glacial and tectonic implications, *Quaternary Science Reviews*, **251**, 106657.

Bradley, S. L., Milne, G. A., Horton, B. P., & Zong, Y., 2016. Modelling sea level data from China and Malay-Thailand to estimate Holocene ice-volume equivalent sea level change, *Quaternary Science Reviews*, **137**, 54–68.

Brunović, D., Miko, S., Hasan, O., Papatheodorou, G., Ilijanić, N., Miserocchi, S., Correggiari, A., & Geraga, M., 2020. Late Pleistocene and Holocene paleoenvironmental reconstruction of a drowned karst isolation basin (Lošinj Channel, NE Adriatic Sea), *Palaeogeography, Palaeoclimatology, Palaeoecology*, **544**, 109587.

Castro, J. W. A., Suguio, K., Seoane, J. C., Cunha, A. M. D., & Dias, F. F., 2014. Sea-level fluctuations and coastal evolution in the state of Rio de Janeiro, southeastern Brazil, *Anais da Academia Brasileira de Ciências*, **86**(2), 671–683.

Chua, S., Switzer, A. D., Li, T., Chen, H., Christie, M., Shaw, T. A., Khan, N. S., Bird, M. I., & Horton, B. P., 2021. A new Holocene sea-level record for Singapore, *The Holocene*, p. 09596836211019096.

Clement, A. J., Whitehouse, P. L., & Sloss, C. R., 2016. An examination of spatial variability in the timing and magnitude of Holocene relative sea-level changes in the New Zealand archipelago, *Quaternary Science Reviews*, **131**, 73–101.

Cooper, J. A. G., Green, A. N., & Compton, J. S., 2018. Sea-level change in southern Africa since the Last Glacial Maximum, *Quaternary Science Reviews*, **201**, 303–318.

Cuffey, K. M., Clow, G. D., Steig, E. J., Buizert, C., Fudge, T. J., Koutnik, M., Waddington, E. D., Alley, R. B., & Severinghaus, J. P., 2016. Deglacial temperature history of West Antarctica, *Proceedings of the National Academy of Sciences*, **113**(50), 14249–14254.

Dalton, A. S., Margold, M., Stokes, C. R., Tarasov, L., Dyke, A. S., Adams, R. S., Allard, S., Arends, H. E., Atkinson, N., Attig, J. W., Barnett, P. J., Barnett, R. L., Batterson, M., Bernatchez, P., Borns, H. W., Breckenridge, A., Briner, J. P., Brouard, E., Campbell, J. E., Carlson, A. E., Clague, J. J., Curry, B. B., Daigneault, R.-A., Dubé-Loubert, H., Easterbrook, D. J., Franzi, D. A., Friedrich, H. G., Funder, S., Gauthier, M. S., Gowan, A. S., Harris, K. L., Hétu, B., Hooyer, T. S., Jennings, C. E., Johnson, M. D., Kehew, A. E., Kelley, S. E., Kerr, D., King, E. L., Kjeldsen, K. K., Knaeble, A. R., Lajeunesse, P., Lakeman, T. R., Lamothe, M., Larson, P., Lavoie, M., Loope, H. M., Lowell, T. V., Lusardi, B. A., Manz, L., McMartin, I., Nixon, F. C., Occhietti, S., Parkhill, M. A., Piper, D. J. W., Pronk, A. G., Richard, P. J. H., Ridge, J. C., Ross, M., Roy, M., Seaman, A., Shaw, J., Stea, R. R., Teller, J. T., Thompson, W. B., Thorleifson, L. H., Utting, D. J., Veillette, J. J., Ward, B. C., Weddle, T. K., & Wright, H. E., 2020. An updated radiocarbon-based ice margin chronology for the last deglaciation of the North American Ice Sheet Complex, *Quaternary Science Reviews*, **234**, 106223.

De Deckker, P. & Yokoyama, Y., 2009. Micropalaeontological evidence for Late Quaternary sea-level changes in Bonaparte Gulf, Australia, *Global and Planetary Change*, **66**(1), 85–92.

Dean, S., Horton, B. P., Evelpidou, N., Cahill, N., Spada, G., & Sivan, D., 2019. Can we detect centennial sea-level variations over the last three thousand years in Israeli archaeological records?, *Quaternary Science Reviews*, **210**, 125–135.

Dechnik, B., Bastos, A. C., Vieira, L. S., Webster, J. M., Fallon, S., Yokoyama, Y., Nothdurft, L., Sanborn, K., Batista, J., Moura, R., & Amado-Filho, G., 2019. Holocene reef growth in the tropical southwestern Atlantic: Evidence for sea level and climate instability, *Quaternary Science Reviews*, **218**, 365–377.

Deiana, G., Lecca, L., Melis, R. T., Soldati, M., Demurtas, V., & Orrù, P. E., 2021. Submarine Geomorphology of the Southwestern Sardinian Continental Shelf (Mediterranean Sea): Insights into the Last Glacial Maximum Sea-Level Changes and Related Environments, *Water*, **13**(2), 155.

Dougherty, A. J., Thomas, Z. A., Fogwill, C., Hogg, A., Palmer, J., Rainsley, E., Williams, A. N., Ulm, S., Rogers, K., Jones, B. G., & Turney, C., 2019. Redating the earliest evidence of the mid-Holocene relative sea-level highstand in Australia and implications for global sea-level rise, *PLOS ONE*, **14**(7), e0218430.

Engelhart, S. E. & Horton, B. P., 2012. Holocene sea level database for the Atlantic coast of the United States, *Quaternary Science Reviews*, **54**, 12–25.

Engelhart, S. E., Vacchi, M., Horton, B. P., Nelson, A. R., & Kopp, R. E., 2015. A sea-level database for the Pacific coast of central North America, *Quaternary Science Reviews*, **113**, 78–92.

Fox-Kemper, B., Hewitt, H.T., Xiao, C., Aalgeirsdóttir, G., Drijfhout, S.S., Edwards, T.L., Golledge, N.R., Hemer, M., Kopp, R.E., Krinner, G., Mix, A., Notz, D., Nowicki, S., Nurhati, I.S., Ruiz, L., Sallée, J.-B., Slangen, A.B.A., & Yu, Y., 2021. 2021: Ocean, cryosphere and sea level change., *The Sixth Assessment Report of the Intergovernmental Panel on Climate Change.*.

Frederikse, T., Landerer, F., Caron, L., Adhikari, S., Parkes, D., Humphrey, V. W., Dangendorf, S., Hogarth, P., Zanna, L., Cheng, L., & Wu, Y.-H., 2020. The causes of sea-level rise since 1900, *Nature*, **584**(7821), 393–397.

Gangadharan, N., Goosse, H., Parkes, D., Goelzer, H., Maussion, F., & Marzeion, B., 2022. Process-based estimate of global-mean sea-level changes in the Common Era, *Earth System Dynamics*, **13**(4), 1417–1435.

García-Artola, A., Stéphan, P., Cearreta, A., Kopp, R. E., Khan, N. S., & Horton, B. P., 2018. Holocene sea-level database from the Atlantic coast of Europe, *Quaternary Science Reviews*, **196**, 177–192.

Garrett, E., Melnick, D., Dura, T., Cisternas, M., Ely, L. L., Wesson, R. L., Jara-Muñoz, J., & Whitehouse, P. L., 2020. Holocene relative sea-level change along the tectonically active Chilean coast, *Quaternary Science Reviews*, **236**, 106281.

Gischler, E., Hudson, J. H., Humblet, M., Braga, J. C., Eisenhauer, A., Isaack, A., Anselmetti, F. S., & Camoin, G. F., 2016. Late Quaternary barrier and fringing reef development of Bora Bora (Society Islands, south Pacific): First subsurface data from the Darwin-type barrier-reef system, *Sedimentology*, **63**(6), 1522–1549.

Gischler, E., Hudson, J. H., Humblet, M., Braga, J. C., Schmitt, D., Isaack, A., Eisenhauer, A., & Camoin, G. F., 2019. Holocene and Pleistocene fringing reef growth and the role of accommodation space and exposure to waves and currents (Bora Bora, Society Islands, French Polynesia), *Sedimentology*, **66**(1), 305–328.

Gray, S. C. & Hein, J. R., 2005. Lagoonal Reef Accretion and Holocene Sea-Level History from Three Atolls in the Cook Islands, Central South Pacific, *Journal of Coastal Research*, pp. 253–264.

Grebennikova, T., Razjigaeva, N., Ganzey, L., Ganzei, K., Arslanov, K., Maksimov, F., Petrov, A., & Kharlamov, A., 2020. Evolution of a paleolake on Russian Island (Sea of Japan) in middle-late Holocene: Record of sea-level oscillations, extreme storms and tsunami, *IOP Conference Series: Earth and Environmental Science*, **438**, 012009.

Hallmann, N., Camoin, G., Eisenhauer, A., Botella, A., Milne, G. A., Vella, C., Samankassou, E., Pothin, V., Dussouillez, P., Fleury, J., & Fietzke, J., 2018. Ice volume and climate changes from a 6000 year sea-level record in French Polynesia, *Nature Communications*, **9**(1), 285.

Hallmann, N., Camoin, G., Eisenhauer, A., Samankassou, E., Vella, C., Botella, A., Milne, G. A., Pothin, V., Dussouillez, P., Fleury, J., Fietzke, J., & Goepfert, T., 2020. Reef response to sea-level and environmental changes in the Central South Pacific over the past 6000 years, *Global and Planetary Change*, **195**, 103357.

Hawkes, A. D., Kemp, A. C., Donnelly, J. P., Horton, B. P., Peltier, W. R., Cahill, N., Hill, D. F., Ashe, E., & Alexander, C. R., 2016. Relative sea-level change in northeastern Florida (USA) during the last ∼8.0 ka, *Quaternary Science Reviews*, **142**, 90–101.

Hibbert, F. D., Rohling, E. J., Dutton, A., Williams, F. H., Chutcharavan, P. M., Zhao, C., & Tamisiea, M. E., 2016. Coral indicators of past sea-level change: A global repository of U-series dated benchmarks, *Quaternary Science Reviews*, **145**, 1–56.

Hibbert, F. D., Williams, F. H., Fallon, S. J., & Rohling, E. J., 2018. A database of biological and geomorphological sea-level markers from the Last Glacial Maximum to present, *Scientific Data*, **5**(1), 180088.

Higley, M., 2000. *Fossil oyster beds of the mid-Holocene highstand of relative sea level: GBR shelf*, Honours thesis, School of Earth Sciences, James Cook University, Townsville, Queensland.

Hijma, M. P. & Cohen, K. M., 2019. Holocene sea-level database for the Rhine-Meuse Delta, The Netherlands: Implications for the pre-8.2 ka sea-level jump, *Quaternary Science Reviews*, **214**, 68–86.

Jones, T. R., Cuffey, K. M., Roberts, W. H., Markle, B. R., Steig, E. J., Stevens, C. M., Valdes, P. J., Fudge, T., Sigl, M., Hughes, A. G.,*et al.*, 2023. Seasonal temperatures in west antarctica during the holocene, *Nature*, **613**(7943), 292–297.

Kaniewski, D., Marriner, N., Cheddadi, R., Morhange, C., Vacchi, M., Rovere, A., Faivre, S., Otto, T., Luce, F., Carre, M.-B., Benčić, G., & Van Campo, E., 2021. Coastal submersions in the north-eastern Adriatic during the last 5200 years, *Global and Planetary Change*, **204**, 103570.

Kemp, A. C., Wright, A. J., Edwards, R. J., Barnett, R. L., Brain, M. J., Kopp, R. E., Cahill, N., Horton, B. P., Charman, D. J., Hawkes, A. D., Hill, T. D., & van de Plassche, O., 2018. Relative sea-level change in Newfoundland, Canada during the past $\sim$3000 years, *Quaternary Science Reviews*, **201**, 89–110.

Kench, P. S., Chan, J., Owen, S. D., & McLean, R. F., 2014. The geomorphology, development and temporal dynamics of Tepuka Island, Funafuti atoll, Tuvalu, *Geomorphology*, **222**, 46–58.

Khadraoui, A., Zaïbi, C., Carbonel, P., Bonnin, J., & Kamoun, F., 2019. Ostracods and mollusks in northern Sfax coast: Reconstruction of Holocene paleoenvironmental changes and associated forcing, *Geo-Marine Letters*, **39**.

Khan, N. S., Ashe, E., Horton, B. P., Dutton, A., Kopp, R. E., Brocard, G., Engelhart, S. E., Hill, D. F., Peltier, W. R., Vane, C. H., & Scatena, F. N., 2017. Drivers of Holocene sea-level change in the Caribbean, *Quaternary Science Reviews*, **155**, 13–36.

Khan, N. S., Horton, B. P., Engelhart, S., Rovere, A., Vacchi, M., Ashe, E. L., Törnqvist, T. E., Dutton, A., Hijma, M. P., & Shennan, I., 2019. Inception of a global atlas of sea levels since the Last Glacial Maximum, *Quaternary Science Reviews*, **220**, 359–371.

Khan, N. S., Ashe, E., Moyer, R. P., Kemp, A. C., Engelhart, S. E., Brain, M. J., Toth, L. T., Chappel, A., Christie, M., Kopp, R. E., & Horton, B. P., 2022. Relative sea-level change in South Florida during the past ˜5 years, *Global and Planetary Change*, p. 103902.

Kirkpatrick, L. H., Green, A. N., & Pether, J., 2019. The seismic stratigraphy of the inner shelf of southern Namibia: The development of an unusual nearshore shelf stratigraphy, *Marine Geology*, **408**, 18–35.

Kopp, R. E., Garner, G. G., Hermans, T. H. J., Jha, S., Kumar, P., Slangen, A. B. A., Turilli, M., Edwards, T. L., Gregory, J. M., Koubbe, G., Levermann, A., Merzky, A., Nowicki, S., Palmer, M. D., & Smith, C., 2023. The Framework for Assessing Changes To Sea-level (FACTS) v1.0-rc: A platform for characterizing parametric and structural uncertainty in future global, relative, and extreme sea-level change, Preprint, Climate and Earth system modeling.

Lambeck, K., Rouby, H., Purcell, A., Sun, Y., & Sambridge, M., 2014. Sea level and global ice volumes from the Last Glacial Maximum to the Holocene, *Proceedings of the National Academy of Sciences*, **111**(43), 15296–15303.

Leclercq, P. W., Oerlemans, J., & Cogley, J. G., 2011. Estimating the Glacier Contribution to Sea-Level Rise for the Period 1800–2005, *Surveys in Geophysics*, **32**(4), 519–535.

Leonard, N. D., Welsh, K. J., Clark, T. R., x. Feng, Y., Pandolfi, J. M., & x. Zhao, J., 2018. New evidence for "far-field" Holocene sea level oscillations and links to global climate records, *Earth and Planetary Science Letters*, **487**, 67–73.

Leorri, E., Fatela, F., Drago, T., Bradley, S. L., Moreno, J., & Cearreta, A., 2013. Lateglacial and Holocene coastal evolution in the Minho estuary (N Portugal): Implications for understanding sea-level changes in Atlantic Iberia, *The Holocene*, **23**(3), 353–363.

Lewis, S. E., Sloss, C. R., Murray-Wallace, C. V., Woodroffe, C. D., & Smithers, S. G., 2013. Post-glacial sea-level changes around the Australian margin: A review, p. 74.

Love, R., Milne, G. A., Tarasov, L., Engelhart, S. E., Hijma, M. P., Latychev, K., Horton, B. P., & Törnqvist, T. E., 2016. The contribution of glacial isostatic adjustment to projections of sea-level change along the Atlantic and Gulf coasts of North America, *Earth's Future*, **4**(10), 440–464.

Mann, T., Bender, M., Lorscheid, T., Stocchi, P., Vacchi, M., Switzer, A. D., & Rovere, A., 2019. Holocene sea levels in Southeast Asia, Maldives, India and Sri Lanka: The SEAMIS database, *Quaternary Science Reviews*, **219**, 112–125.

Marriner, N., Flaux, C., Morhange, C., & Kaniewski, D., 2012. Nile Delta's sinking past: Quantifiable links with Holocene compaction and climate-driven changes in sediment supply?, *Geology*, **40**(12), 1083–1086.

Marzeion, B., Kaser, G., Maussion, F., & Champollion, N., 2018. Limited influence of climate change mitigation on short-term glacier mass loss, *Nature Climate Change*, **8**(4), 305–308.

Miklavič, B., Yokoyama, Y., Urata, K., Miyairi, Y., & Kan, H., 2018. Holocene relative sea level history from phreatic overgrowths on speleothems (POS) on Minami Daito Island, Northern Philippine Sea, *Quaternary International*, **471**, 359–368.

Milne, G. A., Long, A. J., & Bassett, S. E., 2005. Modelling Holocene relative sea-level observations from the Caribbean and South America, *Quaternary Science Reviews*, **24**(10), 1183–1202.

Nott, J., 1996. Late Pleistocene and Holocene Sea-Level Highstands in Northern Australia, *Journal of Coastal Research*, **12**(4), 907–910.

O'Regan, M., Coxall, H., Hill, P., Hilton, R., Muschitiello, F., & Swärd, H., 2018. Early Holocene sea level in the Canadian Beaufort Sea constrained by radiocarbon dates from a deep borehole in the Mackenzie Trough, Arctic Canada, *Boreas*, **47**(4), 1102–1117.

Peltier, W. R., Argus, D. F., & Drummond, R., 2015. Space geodesy constrains ice age terminal deglaciation: The global ICE-6G_C (VM5a) model, *Journal of Geophysical Research: Solid Earth*, **120**(1), 450–487.

Pleuger, E., Goiran, J. P., Mazzini, I., Delile, H., Abichou, A., Gadhoum, A., Djerbi, H., Piotrowska, N., Wilson, A., Fentress, E., Ben Jerbania, I., & Fagel, N., 2019. Palaeogeographical and palaeoenvironmental reconstruction of the Medjerda delta (Tunisia) during the Holocene, *Quaternary Science Reviews*, **220**, 263–278.

Prieto, A. R., Mourelle, D., Peltier, W. R., Drummond, R., Vilanova, I., & Ricci, L., 2017. Relative sea-level changes during the Holocene in the Río de la Plata, Argentina and Uruguay: A review, *Quaternary International*, **442**, 35–49.

Punwong, P., 2013. *Holocene Mangrove Dynamics and Sea Level Changes: Records from the Tanzanian Coast*, Ph.D. thesis, University of York.

Punwong, P., Marchant, R., & Selby, K., 2013. Holocene mangrove dynamics from Unguja Ukuu, Zanzibar, *Quaternary International*, **298**, 4–19.

Punwong, P., Selby, K., & Marchant, R., 2018. Holocene mangrove dynamics and relative sea-level changes along the Tanzanian coast, East Africa, *Estuarine, Coastal and Shelf Science*, **212**, 105–117.

Rashid, T., Suzuki, S., Sato, H., Monsur, M., & Saha, S., 2013. Relative sea-level changes during the Holocene in Bangladesh, *Journal of Asian Earth Sciences*, **64**, 136–150.

Ratnayake, A. S., Sampei, Y., Ratnayake, N. P., & Roser, B. P., 2017. Middle to late Holocene environmental changes in the depositional system of the tropical brackish Bolgoda Lake, coastal southwest Sri Lanka, *Palaeogeography, Palaeoclimatology, Palaeoecology*, **465**, 122–137.

Rehfeld, K. & Kurths, J., 2014. Similarity estimators for irregular and age-uncertain time series, *Climate of the Past*, **10**(1), 107–122, Publisher: Copernicus GmbH.

Rehfeld, K., Marwan, N., Heitzig, J., & Kurths, J., 2011. Comparison of correlation analysis techniques for irregularly sampled time series, *Nonlinear Processes in Geophysics*, **18**(3), 389–404, Publisher: Copernicus GmbH.

Reynolds, L. C. & Simms, A. R., 2015. Late Quaternary relative sea level in Southern California and Monterey Bay, *Quaternary Science Reviews*, **126**, 57–66.

Runds, M. J., Bordy, E. M., & Pether, J., 2019. Late Quaternary sedimentological history of a submerged gravel barrier beach complex, southern Namibia, *Geo-Marine Letters*, **39**(6), 469–491.

Rushby, G. T., Richards, G. T., Gehrels, W. R., Anderson, W. P., Bateman, M. D., & Blake, W. H., 2019. Testing the mid-Holocene relative sea-level highstand hypothesis in North Wales, UK, *The Holocene*, **29**(9), 1491–1502.

Salas-Saavedra, M., Dechnik, B., Webb, G. E., Webster, J. M., Zhao, J.-x., Nothdurft, L. D., Clark, T. R., Graham, T., & Duce, S., 2018. Holocene reef growth over irregular Pleistocene karst confirms major influence of hydrodynamic factors on Holocene reef development, *Quaternary Science Reviews*, **180**, 157–176.

Shackleton, S., Menking, J. A., Brook, E., Buizert, C., Dyonisius, M. N., Petrenko, V. V., Baggenstos, D., & Severinghaus, J. P., 2021. Evolution of mean ocean temperature in Marine Isotope Stage 4, *Climate of the Past*, **17**(5), 2273–2289.

Sloss, C. R., Murray-Wallace, C. V., & Jones, B. G., 2007. Holocene sea-level change on the southeast coast of Australia: A review, *The Holocene*, **17**(7), 999–1014.

Song, B., Yi, S., Yu, S.-Y., Nahm, W.-H., Lee, J.-Y., Lim, J., Kim, J., Yang, Z., Han, M., Jo, K.-N., & Saito, Y., 2018. Holocene relative sea-level changes inferred from multiple proxies on the west coast of South Korea, *Palaeogeography, Palaeoclimatology, Palaeoecology*, **496**.

Ta, T. K. O., Nguyen, V. L., Saito, Y., Gugliotta, M., Tamura, T., Nguyen, T. M. L., Truong, M. H., & Bui, T. L., 2021. Latest Pleistocene to Holocene stratigraphic record and evolution of the Paleo-Mekong incised valley, Vietnam, *Marine Geology*, **433**, 106406.

Tam, C.-Y., Zong, Y., bin Hassan, K., bin Ismal, H., binti Jamil, H., Xiong, H., Wu, P., Sun, Y., Huang, G., & Zheng, Z., 2018. A below-the-present late Holocene relative sea level and the glacial isostatic adjustment during the Holocene in the Malay Peninsula, *Quaternary Science Reviews*, **201**, 206–222.

Tanigawa, K., Hyodo, M., & Sato, H., 2013. Holocene relative sea-level change and rate of sea-level rise from coastal deposits in the Toyooka Basin, western Japan, *The Holocene*, **23**(7), 1039–1051.

Vacchi, M., Ghilardi, M., Melis, R. T., Spada, G., Giaime, M., Marriner, N., Lorscheid, T., Morhange, C., Burjachs, F., & Rovere, A., 2018. New relative sea-level insights into the isostatic history of the Western Mediterranean, *Quaternary Science Reviews*, **201**, 396–408.

Vacchi, M., Ghilardi, M., Stocchi, P., Furlani, S., Rossi, V., Buosi, C., Rovere, A., & De Muro, S., 2020. Driving mechanisms of Holocene coastal evolution in the Bonifacio Strait (Western Mediterranean), *Marine Geology*, **427**, 106265.

Vacchi, M., Joyse, K. M., Kopp, R. E., Marriner, N., Kaniewski, D., & Rovere, A., 2021. Climate pacing of millennial sea-level change variability in the central and western Mediterranean, *Nature Communications*, **12**(1), 4013.

Walker, J. S., Kopp, R. E., Little, C. M., & Horton, B. P., 2022. Timing of emergence of modern rates of sea-level rise by 1863, *Nature Communications*, **13**(1), 966.

Woodroffe, C. D., McGregor, H. V., Lambeck, K., Smithers, S. G., & Fink, D., 2012. Mid-Pacific microatolls record sea-level stability over the past 5000 yr, *Geology*, **40**(10), 951–954.

Woodroffe, S. A., 2009. Testing models of mid to late Holocene sea-level change, North Queensland, Australia, *Quaternary Science Reviews*, **28**(23), 2474–2488.

Xiong, H., Zong, Y., Qian, P., Huang, G., & Fu, S., 2018. Holocene sea-level history of the northern coast of South China Sea, *Quaternary Science Reviews*, **194**, 12–26.

Xiong, H., Zong, Y., Li, T., Long, T., Huang, G., & Fu, S., 2020. Coastal GIA processes revealed by the early to middle Holocene sea-level history of east China, *Quaternary Science Reviews*, **233**, 106249.

Yamano, H., Kayanne, H., Yamaguchi, T., Inoue, T., Mochida, Y., & Baba, S., 2017. Revisiting late Holocene sea-level change from the Gilbert Islands, Kiribati, west-central Pacific Ocean, *Quaternary Research*, **88**(3), 400–408.

Yamano, H., Inoue, T., Adachi, H., Tsukaya, K., Adachi, R., & Baba, S., 2019. Holocene sea-level change and evolution of a mixed coral reef and mangrove system at Iriomote Island, southwest Japan, *Estuarine, Coastal and Shelf Science*, **220**, 166–175.

Yokoyama, Y., Maeda, Y., Okuno, J., Miyairi, Y., & Kosuge, T., 2016. Holocene Antarctic melting and lithospheric uplift history of the southern Okinawa trough inferred from mid- to late-Holocene sea level in Iriomote Island, Ryukyu, Japan, *Quaternary International*, **397**, 342–348.

Zhang, Y., Zong, Y., Xiong, H., Li, T., Fu, S., Huang, G., & Zheng, Z., 2021. The middle-to-late Holocene relative sea-level history, highstand and levering effect on the east coast of Malay Peninsula, *Global and Planetary Change*, **196**, 103369.

Zong, Y., 2004. Mid-Holocene sea-level highstand along the Southeast Coast of China, *Quaternary International*, **117**(1), 55–67.

Zwartz, D., 1995. *The recent history of the Antarctic ice sheet : constraints from sea-level change*, Ph.D. thesis, The Australian National University.
